# Quality Evaluation of Packaged Pimento-Paste Stuffed Green Manzanilla Olives in KCl, CaCl_2_, and MgCl_2_ Brines

**DOI:** 10.3390/foods15010104

**Published:** 2025-12-29

**Authors:** Antonio López-López, José María Moreno-Baquero, Antonio Garrido-Fernández

**Affiliations:** Instituto de la Grasa (IG), Spanish National Research Council (CSIC), Campus Universitario Pablo de Olavide, Edificio 46, Ctra. Utrera km 1, 41013 Sevilla, Spain; jose.moreno.baquero@gmail.com (J.M.M.-B.); agarrido@ig.csic.es (A.G.-F.)

**Keywords:** pimiento-paste stuffed olives, calcium, potassium, magnesium, physicochemical characteristics

## Abstract

This study evaluates the impact of fortifying pimento-paste stuffed olives with nutritional chloride salts (KCl, CaCl_2_, and MgCl_2_) on the quality characteristics of the products, using a D-optimal mixture design and Response Surface Methodology. To this aim, the effects of varying salt concentrations on physicochemical characteristics and sensory descriptors were analysed. Results showed that KCl was predominantly favourable, improving sensory attributes and contributing positively to overall product quality. MgCl_2_ exhibited a neutral influence on most characteristics. However, CaCl_2_, while enhancing *kinaesthetic* properties such as *hardness*, *crunchiness*, and *fibrousness*, also increased bitterness, which may detract from consumer preference. The optimal formulation was identified, highlighting the beneficial effects of KCl and a balanced mixture of CaCl_2_ and MgCl_2_. KCl and MgCl_2_ are key components for fortification. The use of CaCl_2_ improves the kinaesthetic properties but requires caution due to its potential to impart a *bitter* taste, which may not be favourable for specific consumers. These findings provide a basis for developing fortified pimento-paste stuffed olives or similar products tailored to consumer preferences.

## 1. Introduction

Table olive production in the Mediterranean area is an old tradition. Technological developments in the second half of the last century, such as mass fermentation or development of pitting, slicing, simultaneous pitting and stuffing olives, or the production of a diversity of stuffing materials, have enabled the increasing variety of presentation forms, such as those stuffed with pimento strips, pimento-paste, anchovy-paste, or virtually any other stuffing material [1]. There are no reliable statistics on the proportion of stuffed olives in global production, although it may represent approximately 15–20% of Spanish-style production. Among the stuffed olives, those containing pimento/anchovy paste are predominant.

As early as 2007, the World Health Organization (WHO) published “Prevention of Cardiovascular Disease: Guidelines for Assessment and Management of Cardiovascular Risks” to reduce disability and premature deaths from coronary heart disease, cerebrovascular disease, and peripheral vascular disease in high-risk people. The emphasis was focused on lifestyle and prophylactic drug therapies [2]. The interest of WHO on these problems was reflected in a subsequent study on “Global health risks: mortality and burden of disease attributable to selected major risks” [3], which established that “the leading global risks for mortality in the world were high blood pressure (responsible for 13% of deaths globally), tobacco use (9%), high blood glucose (6%), physical inactivity (6%), and overweight and obesity (5%)”. These risks are responsible for the rising risk of chronic diseases such as heart disease, diabetes, and cancer. As a result of this study, the WHO produced “*Guideline: Sodium Intake for Adults and Children*” to provide recommendations on the consumption of sodium to reduce noncommunicable diseases (NCDs) in most adults and children, with a strong recommendation to reduce sodium intake to less than 2 g/day and salt to less than 5 g/day in adults [4]. Since then, many organisations have established plans to reduce salt intake at regional or national levels, following these recommendations [5,6]. In Spain, the action to reduce salt was achieved through the “*Spanish Strategy for Nutrition, Physical Activity and Prevention of Obesity* (NAOS strategy)” [7], which focused mainly on bread, the most salt-rich of the frequently ingested products. The strategy successfully reduced salt by reformulating bread, cereals, and ready meals [8].

Table olives are among the major fermented vegetables in Western countries, with a global annual production of about 3000 million tons. Salt plays a key role in their fermentation and packaging [1,9], and is a relevant source of salt consumption for certain sectors of the population, mainly in the Mediterranean and producer countries. Green Spanish-style stuffed olives represent a substantial proportion of the overall production and are currently packaged under relatively mild (GMP) physicochemical conditions [9] and stabilized by pasteurization [1]. However, they still contain a significant amount of salt, and frequently other savory compounds, such as sodium glutamate, can also contribute to the final products’ sodium content. Contents of sodium in the U.S. Department of Agriculture (USDA) for pimento-paste stuffed olives may range between 800 and 1700 mg/100 g, reaching similar values in olives stuffed with anchovy (1600 mg/100 g), feta cheese (1800 mg/100 g), or garlic (1430 mg/100 g) [10]. So, they can substantially contribute to consumer salt intake, and a reduction in their content aligns with current trends in food salt content.

Reducing salt in foods is important because excessive sodium intake poses health risks. However, other nutrients, such as potassium, calcium, and magnesium, have significant beneficial effects [11,12,13]. Their essential roles in human health highlight the potential advantages of fortifying table olives with these nutrients.

Evidence suggests that increasing potassium intake can effectively lower blood pressure in adults. Potassium naturally occurs in many unprocessed foods, including beans and peas, nuts, vegetables such as spinach, cabbage, and parsley, and fruits like bananas, papayas, and dates [11]. However, table olive processing, like many food processing methods, reduces potassium content. Diets high in processed foods and low in fresh fruits and vegetables are often deficient in potassium. WHO recommends increasing potassium intake from food sources to help lower blood pressure and reduce the risk of cardiovascular disease, stroke, and coronary heart disease. For adults, the WHO recommends a minimum daily potassium intake of 3510 mg/day [14].

Although research on calcium has traditionally focused on its role in bone health, recent studies have also explored its effects on other health outcomes. Adequate calcium intake has been associated with several benefits, including lower risk of hypertensive disorders during pregnancy, reduced blood pressure (especially in young people), prevention of osteoporosis and colorectal adenomas, decreased cholesterol levels, and better blood pressure in children whose mothers consume enough calcium during pregnancy [12,15]. The recommended daily calcium intake for adults is 1000 mg for men and 1200 mg for women [16].

Most of the magnesium is stored in the bones and soft tissues. Less than 1% is found in the blood, where levels are tightly regulated. Magnesium is essential for many biological processes, including energy production, oxidative phosphorylation, glycolysis, DNA and RNA synthesis, and glutathione production. It also plays a vital role in the active transport of calcium and potassium ions across cell membranes, which is crucial for nerve impulse conduction, muscle contraction, and the maintenance of a regular heartbeat [17]. The recommended daily magnesium intake for adults ranges from 310 mg/day (for females) to 420 mg/day (for males) [18].

Although table olives were not included in the “*Spanish Strategy for Nutrition, Physical Activity and Prevention of Obesity* (NAOS strategy)”, several investigations have been conducted to reduce salt during the fermentation of Spanish cultivars [19] and those from other countries [20,21]. Similar fortification efforts have been applied to various fermented products, such as pickles, where potassium or calcium has been used to replace sodium and improve hypertension-related health outcomes [22,23]. However, changes during fermentation may cause undesirable spoilage or safety risks [24]. An alternative to salt reduction during the packaging of green Manzanilla Spanish-style or natural olives has recently emerged [25,26,27], but the effect of fortifying stuffed olives with nutrient-rich salt mixtures on their quality has never been investigated.

This study aimed to examine the effects of salt mixture brines on the physicochemical properties of brines as well as colour, sensory attributes, and consumer evaluation of pimento-paste stuffed green olives, using Response Surface Methodology (RSM). The brines included KCl, CaCl_2_, and MgCl_2_, with the total salt concentration limited to 5%. The sodium level was set at 2.5%, and the remaining 2.5% was distributed among the other salts using a simple D-optimum mixture design. Statistical analysis was performed to develop functional models relating salt concentrations to measured variables and to assess the model’s characteristics.

## 2. Materials and Methods

### 2.1. Olives

Olives of the Manzanilla cultivar were collected at the so-called green maturation stage in September 2012. The fruits were processed during the 2012–2013 season in the traditional Spanish style. After fermentation, the olives were stuffed with pimento-paste in April 2013 and stored in a new brine (approximately 7.5% salt). Then, a fraction of this product (30 kg) was supplied by JOLCA SCA (Huevar, Sevilla, Spain) and stored in a cold room at 8 ± 1 °C at the Instituto de la Grasa pilot plant until use.

### 2.2. Experimental Design

Before packaging, the stuffed olives were desalted in the same cold room (8 ± 1 °C) until the salt level in the olive pulp reached 2.5% sodium chloride (NaCl). This was done by adjusting the amounts of olive and tap water to achieve the desired balance.

Following desalting, the reduced-salt olives were packaged in glass containers (170 g olives/130 mL brine) using different brines. The brine solutions consisted of a mixture of KCl, MgCl_2_, and CaCl_2_, constrained to 25 g/L (Table 1). The experimental design was generated using Design-Expert 13.0 (Stat-Ease Inc., Minneapolis, MN, USA). The listed values correspond to anhydrous salt concentrations targeted for each salt after packaging. These amounts were readjusted to account for the hydration state and olive-to-brine ratio in the container. Regardless of the chloride salt combination, all brines contained 2.5% NaCl to ensure that the olive flesh reached the desired equilibrium salt level, as table olives are traditionally associated with a salty taste. Moreover, previous studies using salt mixtures have shown that completely removing NaCl reduced product acceptability [19]. Consequently, the final concentration of total salts in the brine was consistently 5%, as the “*Trade Standard for Table Olives*” recommends for lye-treated green table olives [9]. To achieve the desired acidity, lactic acid was added to the brine to yield a 0.5% (*w*/*v*) concentration and a pH of approximately 4.0 in the packaged olives. Finally, the glass containers were pasteurized at 85 °C for 8.5 min to reach a ${PU}_{62.4{}^{\circ}C}^{5.25}\geq$ 15, simulating the stabilization process currently applied in the industry. The packaged olives were then stored at 20 ± 2 °C in the pilot plant facilities of the Instituto de la Grasa (Sevilla, Spain) for two months to simulate equilibrium and shelf-life conditions.

### 2.3. Physicochemical Analysis of Fruits

The brine pH, titratable acidity, and combined acidity were measured according to the methods outlined by Garrido-Fernández et al. [1]. Moisture content was determined by drying an aliquot of olive flesh samples on stainless steel plates until a constant weight was reached in a Selecta electric oven at 106 °C (Dry-Big 2002970, Abrera, Barcelona, Spain). Lactic acid concentrations were measured by HPLC [27].

The objective firmness was measured using a Kramer shear compression cell connected to a universal testing machine (Instron, Canton, MA, USA). The cross-head speed was set at 200 mm/min. Firmness values were calculated as the average of 20 measurements, each from one pitted fruit. The shear compression force was expressed in Newtons per gram (N/g).

Olive surface color was measured using a Colour-View spectrophotometer (model 9000, BYK-Gardner, Columbia, MD, USA) equipped with software to calculate the CIE coordinates: L* (lightness), a* (negative values indicate green and positive values indicate red), and b* (negative values indicate blue and positive values indicate yellow) with a C type illuminant at 10°. To minimize stray-light interference, the samples were covered with a box with a matte-black interior. Each measurement was the average of 20 readings from individual olives. Additional color measurements included the ratio −a*/b* (a form of internal standardization), chroma (Ch*) [28,29], and color index (CI), calculated as CI = (−2R_560_ + R_590_ + 4R_635_)/3 [30]. A detailed description of this methodology can be found in López-López et al. [25].

### 2.4. Sensory Analysis

The sensory analysis was conducted in individual booths under controlled conditions of light, temperature, and humidity by 100 IG staff members, regardless of scale, excluding those from the Food Biotechnology Department. All participants were habitual olive consumers [31]. The evaluation form was based on relevant literature [32,33], the “*Sensory Analysis for Table Olives*” [34], and previous experience [35]. Olive samples from the experimental design treatments were introduced in cups, labelled with three randomly selected letters. Then, the cups were randomly presented to testers, who scored each question in the evaluation sheet on a 10 cm unstructured scale. More details on the procedure can be found elsewhere [25].

### 2.5. Data Analysis

The effect of reduced NaCl and the fortified mixtures of KCl, MgCl_2_, and CaCl_2_ brines on the physicochemical and sensory properties of packaged table olives was examined using response surface methodology (RSM), with treatment conditions developed using Design-Expert v13.0 (Stat-Ease Inc., MN, USA). Only terms whose incorporation significantly increased the explained variance were retained. Relevant parameters for assessing fit (R-squared, RMSE, F-value, *p*-value, lack of fit, and precision) are provided for every model. Details of the method and its application can be found elsewhere [25].

### 2.6. Multivariate Analysis

Cluster analysis and PLS were used to examine the relationships between physicochemical parameters and treatments. The methods included correlation, Hierarchical Cluster Analysis (HCA) [36], and Partial Least Squares Regression (PLS-R) [37,38]. The analysis was conducted using XLSTAT v 2017 (Stat-Soft, Paris, France).

## 3. Results

### 3.1. Characterisation of the Raw Material

A detailed description of the raw materials is provided in Table 2. The olives averaged 282 fruits/kg, with individual fruit weights of 3 g. Their size distribution was very uniform, with 95% of the olives ranging from 3.01 to 3.30 g. These pimento-paste stuffed olives showed only a 2 mm difference between diameter and height, giving them a more spherical shape than whole olives [25]. The olives represented 84.20% of the total product. The stuffing material accounted for 15.77%, which is similar to the pit proportion in whole olives (15.42%). However, the stuffing material had a higher density (1.49 g/mL) than the pit (1.37 g/mL) [25]. Plain olives had a density of 1.03 g/mL, while stuffed olives ranged from 0.975 to 1.03 g/mL, with an average of 0.999 g/mL. This density contrast allows the correct separation of properly stuffed olives from whole olives or defective fruits containing pit fragments by flotation [1]. The packaged stuffed olives had an average moisture content of 72.96%. The olives alone showed an average value of 68.49%, while the stuffing material reached 86.90%.

### 3.2. Effect of the Desalting Operation on Key Physicochemical Characteristics of Brines and Fruits

The desalting process leached the NaCl from the olives into the tap water, reaching equilibrium at a target concentration of 25 g/L in the moisture. This process also affected several other parameters (Table 3).

Titratable acidity decreased sharply from 9.20 (SE, 0.40) g/L to 2.95 (0.25) g/L. Lactic acid concentration showed a similar decline, dropping from 17.45 (0.29) g/L to 5.97 (0.05) g/L (Table 3). To compensate for this loss, lactic acid should be also added to the brine during packaging to achieve the recommended 5 g/L in the packaged product, as per IOC guidelines [9].

The pH of the desalting solution changed minimally, increasing by only 0.03 units. In contrast, combined acidity decreased markedly, from 102 (3) mEq/L to 37 (1) mEq/L. This reduction contributed to lower pH in the final products, thereby improving stability. The combined effect of reducing combined acidity during desalting and subsequent dilution during packaging strengthened product preservation.

The lactic acid concentration estimated from titratable acidity (assuming lactic acid as the primary contributor to combined acidity) closely matched the total lactic acid measured by HPLC. This agreement indicates that HPLC quantifies both the titratable acidity and the combined acidity fractions of the lactic acid.

Several fruit characteristics were affected to different degrees during desalting (Table 4). Firmness decreased slightly from 16 (5) N/g in stored olives to 14 (5) N/g after desalting. In contrast, the reduction in lactic acid in the flesh moisture was pronounced and significant, falling from 18 (0.3) g/L to about 7 (0.1) g/L. Flesh moisture increased modestly from roughly 73% (0.5) to around 76% (0.07). This increase corresponded to a weight gain of about 3%.

### 3.3. The Effect of Packaging in KCl, CaCl_2_, and MgCl_2_ Fortified Brines on Key Physicochemical Characteristics of Brine

Using salt mixtures in the packaging brines also caused variations in the physicochemical parameters of the experimental treatments. In the brines, the pH increased from 3.30 to 3.94. Titratable acidity ranged from 2.05 to 2.50 g/L. Combined acidity spanned 20.9 to 24.8 mEq/L, and estimated lactic acid levels fluctuated between 4.28 and 4.56 g/L. Among these parameters, only combined acidity showed a significant relationship with the salt mixture concentrations in the packaging brine.

**Figure 1:**
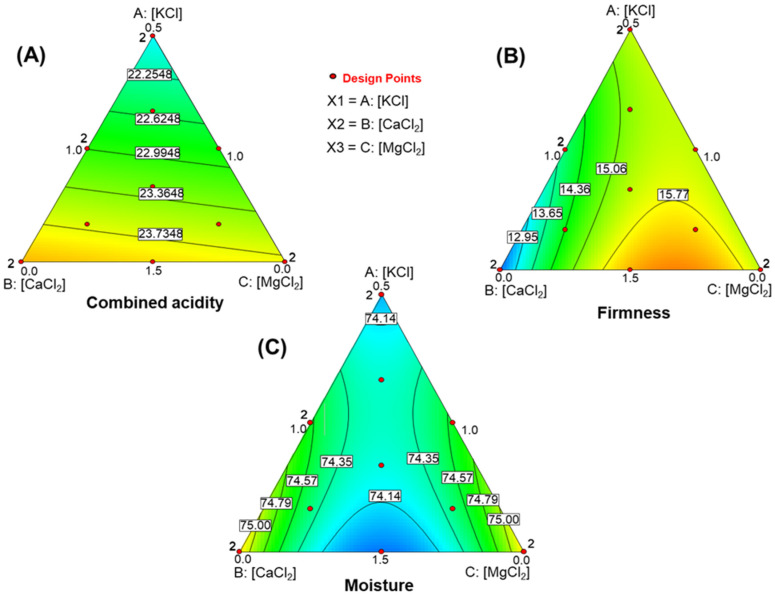
Effects of KCl, CaCl_2_, and MgCl_2_-enriched brines on quality traits of packaged pimento-paste stuffed green Spanish-style olives. (**A**) Combined acidity, (**B**) Firmness, (**C**) Moisture.

The model predicting combined acidity was significant (R^2^ = 0.6209, RMSE = 0.654, F = 9.01, *p* = 0.0048). Lack of fit was non-significant (*p* = 0.8629), and the model showed adequate precision (7.542), well above the threshold of 4 for a strong signal-to-noise ratio. These results indicate that the model (Figure 1A) is robust and suitable for exploring the experimental design space. The resulting equation was linear and had the following form:

$$Combined\;acidity\;(mEq/L)\;=+\;1.040\cdot \left[KCl\right]+0.818\cdot \left[{CaCl}_{2}\right]+0.850\cdot [{MgCl}_{2}]$$

Potassium salt appears to be the main factor influencing combined acidity in the experimental runs. However, the function is more effectively interpreted by examining its simplex plot. Combined acidity increases as the formulation moves farther from the KCl vertex, which corresponds to the lowest potassium salt level (5 g/L). The plot also shows a slightly steeper gradient—visible as wider contour spacing—toward the MgCl_2_ vertex. This indicates that increasing MgCl_2_ is somewhat more effective than increasing CaCl_2_ in lowering combined acidity.

### 3.4. The Effect of Packaging in KCl, CaCl_2_, and MgCl_2_ Fortified Brines on Key Physicochemical Characteristics of Pimento-Paste Stuffed Olives

Firmness values in the Control were consistently lower than those in any of the design treatments. In contrast, the moisture and lactic acid levels were close to the average of the design runs.

The effect of fortified salts on firmness, moisture, and lactic acid levels in the flesh had different degrees. Firmness ranged from 11.63 to 17.18 N/g. Moisture varied between 73.69 to 75.63 g/100 g of flesh. Lactic acid in the product moisture ranged from 4.31 to 4.44 g/L. These parameters showed considerable variability across runs, especially firmness and moisture.

Although lactic acid concentrations in the packaged stuffed-olive moisture were lower than their values after desalting (Table 4), they remained similar to those in the packaging brine (Table 3). This indicates that, when estimating lactic acid concentrations at equilibrium, only moisture content should be considered—not the total mass of stuffed olives.

The initial salt mixture concentrations in the packaging brines significantly impacted all parameter values, revealing notable relationships, discussed below.

#### 3.4.1. Firmness

The firmness model significance (R^2^ = 0.6277, RMSE = 0.525, F = 5.62, *p* = 0.0161). The lack of fit was not significant (*p* = 0.1360), and model precision was high (6678). Thus, the model is suitable for navigating the experimental region (Figure 1B). The model equation was:

$$Firmness\;\left(\frac{N}{g}\right)=+\;0.870\cdot \left[KCl\right]+0.233\cdot \left[{CaCl}_{2}\right]-0.081\cdot \left[{MgCl}_{2}\right]\;+0.096\cdot \left[{CaCl}_{2}\right]\cdot \left[{MgCl}_{2}\right]$$

KCl and CaCl_2_ appear to have the strongest influence, based on their larger coefficients, but the relationship is not straightforward. The simplex plot (Figure 1B) shows a gradual improvement in firmness as the CaCl_2_ concentration increases. The highest firmness occurs at the maximum KCl levels (15 g/L), combined with roughly two-thirds of the CaCl_2_ range (7.5 g/L) and one-third of the MgCl_2_ range (3.3 g/L). This pattern suggests an interaction between Ca^2+^ and Mg^2+^, likely related to their similar ionic charges, while optimal firmness still requires high KCl concentrations.

#### 3.4.2. Moisture in Pimento-Paste Stuffed Olive

The moisture model was significant (R^2^ = 0.5893, RMSE = 0.292, F = 4.78, *p* = 0.0256). The lack of fit was not significant (*p* = 0.8026), and model precision was acceptable (5.439). Therefore, the model is suitable for navigating within the design space (Figure 1C). The equation was:

$$\;\;\;\;\;Moisture\;\left(g/100g\;flesh\right)=\;2.851\cdot \left[KCl\right]+3.246\cdot \left[{CaCl}_{2}\right]+3.241\cdot \left[{MgCl}_{2}\right]-0.051\cdot \left[{CaCl}_{2}\right]\cdot [{MgCl}_{2}]$$

The simplex plot (Figure 1C) indicates that KCL has little influence on moisture in the central region. Moisture increases when CaCl_2_ and MgCl_2_ levels exceed one-third of their respective ranges. Moisture is lowest when both CaCl_2_ and MgCl_2_ are at their maximum, which corresponds to the KCl vertex.

The effect of CaCl_2_ and MgCl_2_ is symmetrical: moisture reaches a maximum when either salt is at its highest levels, and the other is absent, with peaks near their respective vertices. Conversely, when CaCl_2_ and MgCl_2_ concentrations are similar—along the line from the KCl vertex to the midpoint of the CaCl_2_-MgCl_2_ edge—KCl has minimal effect. Moisture values at the KCl vertex (5 g/L) and along the opposite CaCl_2_-MgCl_2_ border remain similar (about 74.14%, *w*/*w*).

#### 3.4.3. Concentration of Lactic Acid in Pimento-Paste Stuffed Olive Moisture

**Figure 2:**
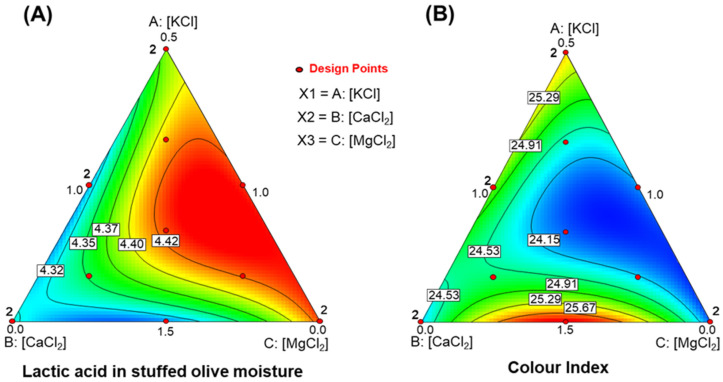
Effects of in KCl, CaCl_2_, and MgCl_2_-enriched brines on quality traits of packaged pimento-paste stuffed green Spanish-style olives. (**A**) Lactic acid in the entire stuffed olive moisture; (**B**) Colour index.

The lactic acid model was significant (R^2^ = 0.8494, RMSE = 0.001, F = 6.58, *p* = 0.0128). Lack of fit was non-significant (*p* = 0.9716), and the model precision was acceptable (6.015). The model was therefore appropriate for navigating the design space (Figure 2A). The equation was:

$$\begin{array}{ll} Lactic\;acid\;(g/L)\; & \\ & =0.074\cdot \left[KCl\right]+0.0067\cdot \left[{CaCl}_{2}\right]-0.0410\cdot \left[{MgCl}_{2}\right]+0.0217\cdot \left[KCl\right]\cdot \left[{CaCl}_{2}\right]+0.024\\ & \cdot \left[KCl\right]\cdot \left[{MgCl}_{2}\right]-0.0023\cdot \left[KCl\right]\cdot \left[{CaCl}_{2}\right]\cdot [{MgCl}_{2}] \end{array}$$

The function is complex and is best interpreted through its simplex plot. Although the absolute effect is small, the plot shows that lactic acid in the stuffed olive moisture tends to increase, reaching its highest levels at about two/thirds of the CaCl_2_ range and at intermediate levels of both KCl and MgCl_2_. This high-lactic region extends towards the MgCl_2_ vertex as MgCl_2_ concentration decreases.

### 3.5. Effect of KCl, CaCl_2_, and MgCl_2_ Fortified Brines on Pimento-Paste Stuffed Olive Colour

The olive is the most visible component of green pimento-paste stuffed olives, because the stuffing material is often largely hidden with flesh removed during stuffing process. No noteworthy visual differences related to the stuffing material were observed between runs, so only the olive colour was analysed (Table 5).

Several parameters were evaluated because colour is a key quality attribute. Desalting led to a slight increase in browning, as reflected in higher L* values and a larger hue angle. These changes were reversed in most treatments, although they persisted in a few cases. The Control samples fell within the range of values observed for experimental treatments.

The ranges of the evaluated colour parameters were: CI, 23.71–26.05; L*, 48.45–50.67; a*, 4.09–4.86; b*, 30.95–32.38; chroma (*Ch*), 31.23–33.11; hue angle (h*), 81.44–92.91; and (−a*/b*), −0.150 to −0.124. All experimental runs fell within the acceptable range based on the colour index developed by Sánchez Gómez et al. [30]. Although all colour parameters showed notable variability, only the colour index (CI) was significantly influenced by salt mixture concentrations.

The model for CI was statistically significant (R^2^ = 0.8805, RMSE = 0.191, F = 8.59, *p* = 0.0060). Lack of fit was non-significant (*p* = 0.8377), and model precision was high (8.553). The resulting equation was:

$$CI=2.720\cdot \left[KCl\right]+3.426\cdot \left[{CaCl}_{2}\right]+4.072\cdot \left[{MgCl}_{2}\right]-0.349\cdot \left[KCl\right]\cdot \left[{CaCl}_{2}\right]-0.382\cdot \left[KCl\right]\cdot \left[{MgCl}_{2}\right]\;-0.455\cdot \left[{CaCl}_{2}\right]\cdot \left[{MgCl}_{2}\right]+0.036\cdot \left[KCl\right]\cdot \left[{CaCl}_{2}\right]\cdot [{MgCl}_{2}]$$

The plot (Figure 2B) highlights a strong correlation between lactic acid in the stuffed olive moisture and the colour index (CI). Higher lactic acid levels corresponded to lower CI values. This finding underscores the complex relationships among table olive characteristics. And highlights the important influence of lactic acid on the colour of pimento-paste stuffed Manzanilla olives.

### 3.6. Effect of Fortified Chloride Salt Brines on the Organoleptic Characteristics, Overall Score, and Consumer Purchasing Predisposition

The olives from different batches were evaluated using a sensory analysis sheet adapted from the IOC’s “*Sensory Analysis of Table Olives*” [34], which additionally included *overall evaluation* and *buying predisposition* [25]. Each response was first checked for negative attributes—*abnormal fermentation*, *musty*, *rancid*, *cooking effect*, *soapy*, *metallic*, *earthy*, and *winey-vinegary*—grouped under “Others”. Scores for these negative attributes consistently averaged around 1, well below the 2.5 threshold for classifying olives as defective. Consequently, all batches were classified as “*First Category*” (I or “*Fancy*”) and deemed suitable for consumption.

After following this pre-classification, the olives were assessed for their actual sensory attributes. Scores for each attribute were measured and recorded, with averages and standard errors summarised in Table 6.

Scores across the different runs were generally consistent. For the experimental treatments, favourable descriptors—*appearance*, *odour*/*smell*, *acid*, *hardness*, *fibrousness*, and *crunchiness*—usually scored above 5. Only two unfavourable descriptors, *bitter* and *salty*, exceeded desirable levels.

Notably, run 4 received an overall evaluation score of 4.64, slightly below 5. This indicates that the combination with the highest CaCl_2_ and MgCl_2_ proportions and the lowest KCl is not recommended. Despite this, the *overall score* of the treatments was encouraging.

Scores for *purchasing predisposition* were less favourable, with several runs ranging from 4.18 to 4.74, slightly below the neutral point. This is not a concern, as the study intentionally used broad limits to capture a wide sensory range. Compared to the Control, only *bitter* (low score) and *purchasing predisposition* (high score) were more favourable, highlighting a strong relationship between these properties.

These results emphasise the importance of carefully selecting salt mixtures for packaging. To improve acceptability, mixtures that minimize bitterness should be prioritized to meet consumer expectations for pimento-paste stuffed olives.

As mentioned, salt concentrations in the packaging brine affected several attributes. Their relationships are described below.

#### 3.6.1. *Bitter*

The model for *bitter* was significant (R^2^ = 0.9163, RMSE = 0.033, F = 60.18, *p* = 0.0001). Lack of fit was non-significant (*p* = 0.7736), and precision was high (20.789). The equation was:

$$Bitter\;=\;+0.185\cdot \left[KCl\right]+0.331\cdot \left[{CaCl}_{2}\right]+0.283\cdot \left[{MgCl}_{2}\right]$$

**Figure 3:**
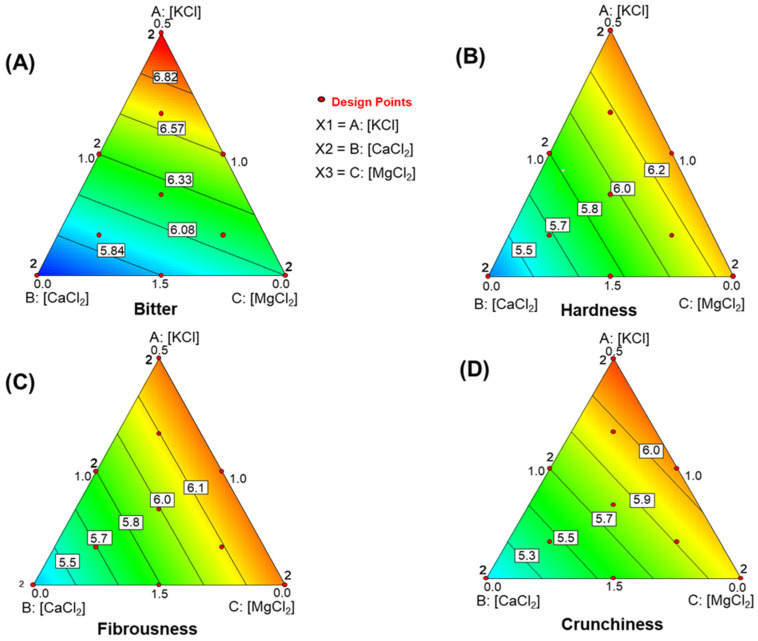
Effects of in KCl, CaCl_2_, and MgCl_2_-enriched brines on sensory attributes of packaged pimento-paste stuffed green Spanish-style olives. (**A**) *Bitter*; (**B**) *Hardness*; (**C**) *Fibrousness*; (**D**) *Crunchiness*.

The largest coefficient was for CaCl_2_, followed by MgCl_2_, consistent with the highest *bitter* score—and the lowest overall evaluation—observed in run 4, which had high levels of these salts. The simplex plot (Figure 3A) shows scores increasing toward the KCl vertex, where KCl is the lowest, and CaCl_2_ and MgCl_2_ are at their highest. Contour lines slope steeply toward the MgCl_2_ vertex, highlighting the predominant influence of CaCl_2_ on this attribute.

#### 3.6.2. *Hardness* (Sensory Evaluation for Firmness)

KCl, CaCl_2_, and MgCl_2_ also influenced *hardness* scores. The model was statistically significant (R^2^ = 0.8167, RMSE = 0.030, F = 24.51, *p* = 0.0001). Lack of fit was non-significant (*p* = 0.4540), and precision was high (12.215).

The model took the form:

$$Hardness\;=\;0.209\cdot \left[KCl\right]+0.313\cdot \left[{CaCl}_{2}\right]+0.219\cdot \left[{MgCl}_{2}\right]$$

The *hardness* function was linear, with CaCl_2_ having the largest coefficient, while KCl and MgCl_2_ contributed almost equally. The simplex plot (Figure 3B) shows that CaCl_2_ has the strongest influence, as the contour lines rise nearly parallel from the CaCl_2_ vertex (0 g/L) to the opposite border (10 g/L). MgCl_2_ caused slightly more interference than KCl, as indicated by the contour lines.

#### 3.6.3. *Fibrousness*

The model for fibrousness was robust (R^2^ = 0.8207, RMSE = 0.023, F = 25.17, *p* = 0.0001). Lack of fit was non-significant (0.4010), and precision was high (11.871). The equation was:

$$Fibrousness\;=\;0.214\cdot \left[KCl\right]+0.306\cdot \left[{CaCl}_{2}\right]+0.215\cdot \left[{MgCl}_{2}\right]$$

The pattern resembled that of *hardness*, as seen in the simplex plot (Figure 3C). Contour lines ran parallel to the KCl-MgCl_2_ side, showing similar contributions from these salts. The similarity between the equations and plots for *hardness* and *fibrousness* suggests that distinguishing between these attributes is difficult. This support simplifies the IOC sensory analysis sheet [31] by combining them.

#### 3.6.4. *Crunchiness*

The model for crunchiness was significant (R^2^ = 0.7224, RMSE = 0.023, F = 14.31, *p* = 0.0009). Lack of fit was non-significant (0.1596), and precision was 9.8866. The model was:

$$Crunchiness\;=\;+0.197\cdot \left[KCl\right]+0.300\cdot \left[{CaCl}_{2}\right]+0.221\cdot \left[{MgCl}_{2}\right]$$

The structure was similar to that for *hardness* and *fibrousness*, and the simplex plot (Figure 3D) showed comparable trends. *Crunchiness* increased with higher CaCl_2_ (from the CaCl_2_ vertex to the KCl-MgCl_2_ border). Contour lines leaned slightly toward the KCl-MgCl_2_ border. Maximum scores occurred with minimal KCl and MgCl_2_, and CaCl_2_ mainly influencing this attribute. Overall, *hardness* (Figure 3B), *fibrousness* (Figure 3C), and *crunchiness* (Figure 3D) follow similar patterns, highlighting their close connection. These results suggest that the interactions between these attributes deserve further research.

#### 3.6.5. *Overall Score*

This attribute was designed to summarize overall impressions into a single value. The model was significant (R^2^ = 0.6812, RMSE = 0.072, F = 7.12, *p* = 0.0076). The lack of fit was non-significant (0.6129), and the precision was reasonable (8.963). The function was:

$$Overall\;score\;=0.354\cdot \left[KCl\right]+0.018\cdot \left[{CaCl}_{2}\right]+0.011\cdot \left[{MgCl}_{2}\right]+0.029\cdot \left[{CaCl}_{2}\right]\cdot \left[{MgCl}_{2}\right]$$

**Figure 4:**
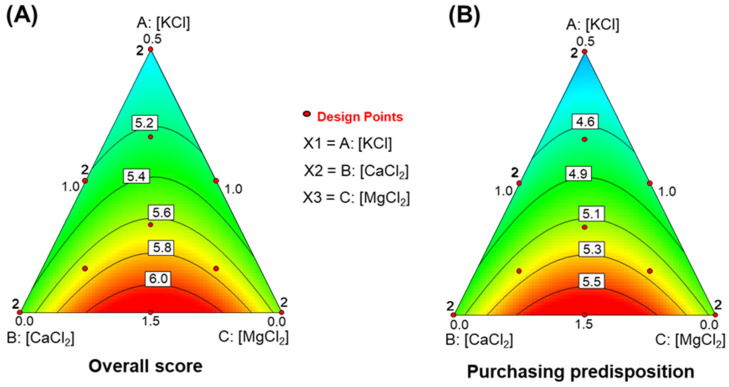
Effects of in KCl, CaCl_2_, and MgCl_2_-enriched brines on *overall* and *purchasing predisposition* of packaged pimento-paste stuffed green Spanish-style olives. (**A**) *Overall score*; (**B**) *Purchasing predisposition*.

KCl made the strongest contribution to this score. The simplex plot (Figure 4A) supports this, showing a steady increase in the *overall score* as KCl concentration in the packaging increases. CaCl_2_ and MgCl_2_ had minor adverse effects, especially when present at similar levels. The response peaks along the vertical line from the KCl vertex to the opposite CaCl_2_-MgCl_2_ border. The highest *overall scores* occurred at the maximum KCl concentration (15 g/L) combined with mid-range levels of CaCl_2_ (5 g/L) and MgCl_2_ (5 g/L).

#### 3.6.6. *Purchasing Predisposition*

This attribute evaluated willingness to purchase the products after testing. The model was significant (R^2^ = 0.6807, RMSE = 0.103, F = 7.11, *p* = 0.0077). Lack of fit was non-significant (0.7292), and precision was 8.951. The function was:

$$Purchasing\;predisposition\;=\;0.346\cdot \left[KCl\right]+0.017\cdot \left[{CaCl}_{2}\right]-0.021\cdot \left[{MgCl}_{2}\right]+0.030\cdot \left[{CaCl}_{2}\right]\cdot \left[{MgCl}_{2}\right]$$

The model structure was similar to that of the *overall score*. The main difference is the negative effect associated with MgCl_2_. The simplex plot (Figure 4B) was also very similar to that of the *overall score*, with slightly lower values.

This pattern suggests that the *overall score* is rated more generously than *purchasing predisposition*. The difference may reflect a more cautious or selective response when purchasing—and therefore costs—is implicitly considered.

However, regarding consumers’ attitudes, it should be noted that the sample used in this study may introduce some bias compared to the general or another specific population. So, translating these results to another context should be made with caution.

### 3.7. Relationships Between Descriptors

The mental balance between positive and negative attributes can influence the *overall score* and *purchasing predisposition*. Correlation analysis is a valuable approach to explore these relationships, and the corresponding coefficients are presented in Table 7.

*Appearance* was significantly correlated with *odour*/*smell*. Acid showed moderate correlations with *salty* and *hardness*, likely due to confusion between *salty* and *acid* sensations or because calcium affects all three attributes. *Bitter* was the most strongly correlated attribute. It was positively associated with *hardness*, *fibrousness*, and *crunchiness*. These links may reflect the fact that all four attributes increase when olives receive a milder lye treatment or because calcium enhances them in a similar way. The three kinaesthetic sensations—*hardness*, *fibrousness*, and *crunchiness*—were highly correlated with one another. This may indicate difficulties when distinguishing between these attributes during tasting.

*Overall score* and *purchasing predisposition* were also strongly correlated. *Bitter* showed a clear negative relationship with both, confirming that *bitter* taste is the least desirable attribute for acceptance. In contrast, *appearance* and *odour*/*smell* were the primary contributors to a higher *overall score* and greater *purchasing predisposition*.

### 3.8. Relationships Between Sensory Characteristics and Overall Score and Purchasing Predisposition Using PLS-R and Cluster Analysis

**Figure 5:**
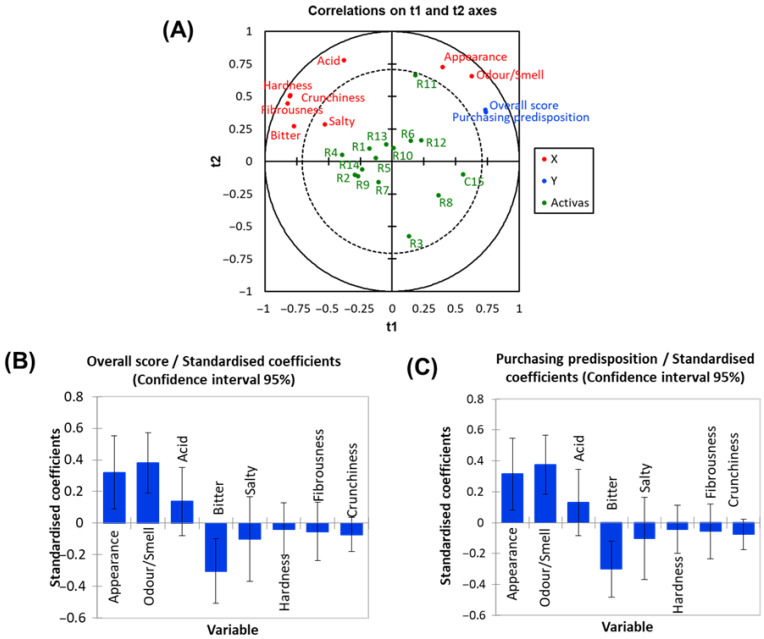
Effects of in KCl, CaCl_2_, and MgCl_2_-enriched brines on quality characteristics of packaged pimento-paste stuffed green Spanish-style olives. (**A**) PLS projection on the t1/t2 plane of sensory attributes, *overall score*, *purchasing predisposition*, and design treatments. (**B**) Standardized coefficients for the *overall score*; (**C**) Standardized coefficients for *purchasing predisposition*.

Partial Least Squares Regression (PLS-R) was also used to evaluate the relationships among sensory attributes and their influence on *overall scores* and purchasing predisposition. A model with four latent variables explained a large proportion of the variation in both the X variable (Q2Xcumm. = 0.741) and the Y variable (Q2Y cumm. = 0.694). The projection on the t1/t2 plane (Figure 5A) shows that t1 clearly separates the sensory attributes.

There was a strong link between the *overall score* and *purchasing predisposition*. Both were positively associated with *odour*/*smell* and *appearance*, and all showed positive correlation with t1. In contrast, the *overall score* and *purchasing predisposition* were negatively related to *bitter*, *hardness*, *fibrousness*, *crunchiness*, *salty*, and acid, all of which correlated negatively with t1. The plot also reinforces the close link between *appearance* and *odour*/*smell*, as well as the strong grouping of *bitterness*, *fibrousness*, *hardness*, and *crunchiness*.

Regarding the treatments, C15, R11, and R8 were positioned closest to the *overall score* and *purchasing predisposition*. Treatments on the right side of the plot were positively associated with t1 and the attributes linked to it. Those on the left were likely characterised by higher scores for the negatively associated attributes and, therefore, showed a negative relationship with t1. In contrast, t2 contributed little to differentiate attribute descriptors and provides only limited separation among treatments, with only R11 and R3 appearing distinct along this axis.

The standardized coefficients offer a clearer view of how each sensory attribute contributes to predicting the *overall score* and *purchasing predisposition* (Figure 5B and C). *Appearance* and *odour*/*smell* showed positive effects, while *bitter* had an adverse effect. These three attributes were the strong contributors to both response variables, reinforcing the close link between the *overall score* and *purchasing predisposition*.

**Figure 6:**
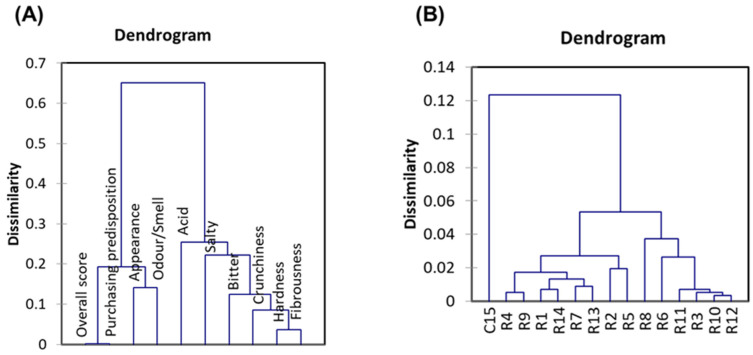
Effects of in KCl, CaCl_2_, and MgCl_2_-enriched brines on quality characteristics of packaged pimento-paste stuffed green Spanish-style olives. (**A**) Relationships between sensory attributes, *overall score*, and *purchasing predisposition*, examined through clustering; (**B**) Connection between treatments based on their sensory attributes, analysed by clustering.

Clustering analysis also helps identify similarities among attributes (Figure 6A) or treatments (Figure 6B), providing a simple and intuitive interpretation. Two main groups of sensory attributes emerged, consistent with the PLS-R analysis. The first group includes the *overall score* and *purchasing predisposition*, which were nearly identical, along with *appearance* and *odour*/*smell.* Although appearance and odour/smell form a tight cluster, their slight separation indicates that they are distinguishable (Figure 6A). The second cluster contains the remaining attributes. Within this group, *acid* and *salty* were separated from *bitter*, but the differences were smaller than those separating the kinaesthetic sensations. *Hardness* and *fibrousness* were the most closely related, and crunchiness was also nearby. This pattern implies that tasters similarly perceive these kinaesthetic sensations, supporting previous findings in table olive studies [25,26].

Regarding treatments, the traditional packaging differed from the experimental packaging, resulting in separate product groups. Among the experimental treatments, two main groups emerged, matching the separation observed in the PLS-R analysis. However, the cluster analysis further refined these distinctions. The NaCl cannot explain this segregation, as all treatments contain similar levels (around 2.5%). Instead, the grouping reflects only the sensory characteristics of the olives. This means that different mineral salt combinations can fall within the same sensory cluster, and each cluster may contain several distinct mineral profiles.

### 3.9. Relationships Between the Sensory Descriptors, the Salt Concentrations in the Packaging Brines, and Physicochemical Characteristics

**Figure 7:**
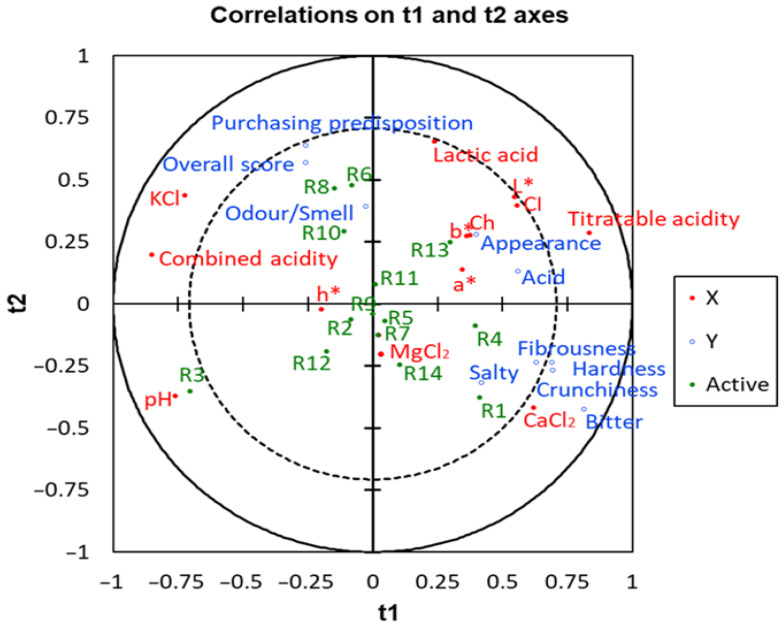
Effects of in KCl, CaCl_2_, and MgCl_2_-enriched brines on quality characteristics of packaging pimento-paste stuffed green Spanish-style olives. Projection on the plane t1/t2 of various physicochemical parameters, sensory attributes, *overall score*, *purchasing predisposition*, and treatments.

The relationships between added salt concentrations, physicochemical properties, and olive colour (predictor variables) with sensory characteristics (the response) were also examined using PLS-R. The selected model, which included three latent variables, revealed several interesting relationships (Figure 7). A strong association was observed between CaCl_2_ concentration and kinaesthetic attributes. CaCl_2_ was also associated with *salty* and *bitter* attributes. Titratable acidity and lactic acid correlated with *colour index*, *appearance*, and *acid*. This pattern was expected, as a good *appearance* generally requires adequate lactic acid and low combined acidity. A notable result was the strong association between the *overall score* and the *purchasing predisposition*, both of which were driven by *odour*/*smell*. This highlights the importance of optimizing *odour*/*smell* to improve acceptance. The presence of KCl may also help to enhance the product’s appeal.

Combined acidity and pH were positioned opposite lactic acid, related to lactic acid and titratable acidity, confirming their inverse relationship: higher lactic acid typically lowers both pH and combined acidity. Finally, MgCl_2_ appeared near the centre of the graph, indicating that it has little influence on either physicochemical or sensory characteristics.

### 3.10. Choosing Optimal Conditions Based on Desirability

RSM facilitates the selection of an optimal salt formulation under defined constraints, which can be adjusted as needed. In this case, the constraints were categorized as follows:

– Variables constrained within the range: mineral salt contents, *hardness*, *fibrousness*, and *crunchiness*;
– Variables to be maximized: titratable acidity, lactic acid, instrumental firmness, *overall score*, and *purchasing predisposition*;
– Variables to be minimized: pH, combined acidity, colour index, moisture in the flesh, *appearance*, *odour*/*smell*, *bitter*, and *salty*.

**Figure 8:**
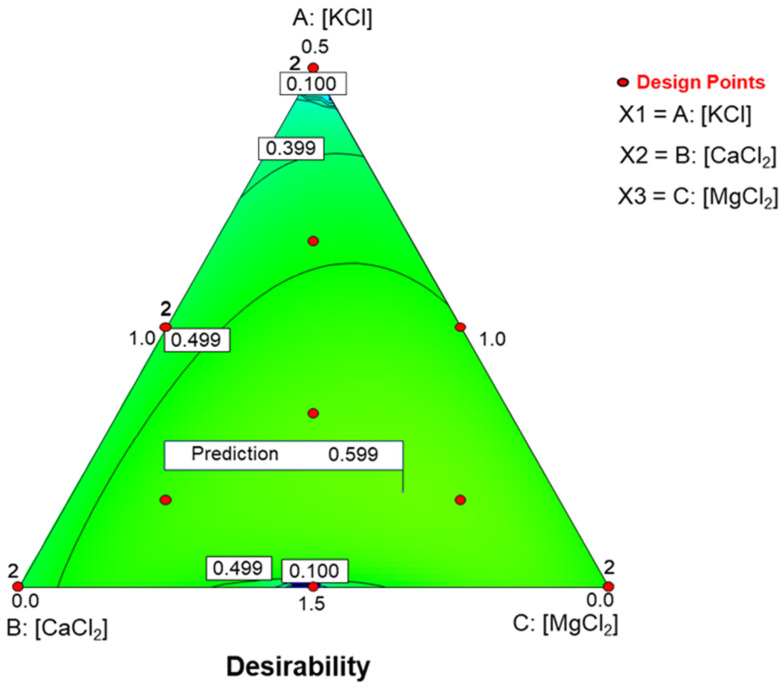
Effects of in KCl, CaCl_2_, and MgCl_2_-enriched brines on quality characteristics of packaged pimento-paste stuffed green Spanish-style olives. Optimization, with the following constraints: mineral salts, *hardness*, *fibrousness*, and *crunchiness*, within the specified range; titratable acidity, lactic acid, instrumental firmness, *overall score*, *purchasing predisposition*, at maximum; pH, combined acidity, colour index, moisture in flesh, *appearance*, *odour*/*smell*, *bitter*, and *salty*, at minimum.

The optimal combination (desirability = 0.599) was obtained with 13.16 g/L of KCl, 7.43 g/L of CaCl_2_, and 4.41 g/L of MgCl_2_. Figure 8 shows this optimal point on simplex plot. The response surface rises with increasing KCl concentration, with a maximum at higher proportions of CaCl_2_ than MgCl_2_, near the indicated point (desirability prediction = 0.599). Beyond this point, desirability decreases, particularly near the CaCl_2_-MgCl_2_ boundary at the highest KCl concentrations and about the same levels of CaCl_2_-MgCl_2_.

The product will have the following characteristics: pH, 3.63; titratable acidity, 2.36 g lactic/L; combined acidity, 23.52 mEq/L; lactic acid (including that as lactate), 4.47 g/L; colour index, 24.26; firmness (instrumental), 15.98 N/g; moisture in the flesh, 74.25%; *appearance*, 6.70; *odour*/*smell*, 6.21; *bitter*, 6.14; *salty*, 6.33; *hardness* (sensory score), 6.00; *fibrousness*, 6.00; *crunchiness*, 5.80; *overall score*, 5.80; *purchasing predisposition*, 5.3.

## 4. Discussion

Reformulating salt use during table olive fermentation entails microbial risks, since the current open-top fiberglass tanks are prone to contamination. Additionally, this modification can result in substantial added mineral losses during conditioning. In contrast, fortifying packaging brines with potassium, calcium, and magnesium in products, although presenting technological challenges, implies a more rational use of salts and reduces spoilage hazard. This study focused on pimento-paste stuffed olives, but the results are relevant to similar commercial products. In these olives, the stuffing replaces the pit. It has about the same weight but higher moisture. The stuffing is often a calcium-enriched gel, which further differentiates stuffed olives as a unique category of table olives.

Response Surface Methodology (RSM) was a key tool in this study. It provides more information than factorial designs with fewer experiments. It is especially suitable when components are not independent, as salt proportions should sum to a fixed total. Classical ANOVA designs are not appropriate under these constraints. RSM also involves the identification of optimal mixtures. However, it has limitations. RSM identifies statistical relationships, but does not explain biochemical mechanisms. It may mask individual effects, and interpretation is less straightforward than in single-factor designs. Outcomes depend on the quality of the fitted model, which may oversimplify nonlinear or threshold effects. Despite these limitations, the D-optimal mixture design was essential to relate salt concentrations in the packaging brine to physicochemical and sensory changes. The resulting equations can also predict salt mixtures that induce specific product characteristics. Nevertheless, caution is needed when using models with moderate explained variance.

Desalting caused a slight increase in pH. This effect resulted from marked reductions in titratable acidity, combined acidity, and estimated lactic acid in the desalting solution. Desalting also increased stuffed olive moisture but only slightly affected firmness.

Packaging decreased pH in the fortified products due to the lactic acid added to the new brine. This effect was consistent across runs and independent of the fortifying minerals. Combined acidity decreased further after packaging due to dilution. This reduction explains the lower pH values in the experimental runs compared to the Control. Combined acidity was influenced by salt composition. Higher proportions of CaCl_2_ and MgCl_2_ led to lower combined acidity, possibly due to the formation of phenol-divalent complexes [39]. The combination of lower combined acidity and pH helps product stability improvement [9]

Reformulating the salt composition of packaging brines also affected other cover brine characteristics. Overall acidity decreased, despite lactic acid addition. Although this effect is rarely reported, similar behaviour has been observed in plain green Spanish-style [25] and natural green table olives [26].

Firmness is clearly influenced by salt composition. Increasing CaCl_2_ levels increased firmness. Maximum firmness required also high KCl levels and intermediate (around 0.33 g/L) MgCl_2_ levels. This pattern aligns with post-harvest olive treatments with calcium chloride sprays (58.5 mM), which produce firmer fruits with lower soluble pectin and higher calcium pectate levels than untreated fruits [40].

The packaging salts modulated changes in firmness and moisture. Firmness was strongly linked to CaCl_2_. Moisture behaviour was more complex. Minimum moisture occurred along the line from the KCl vertex to the opposite CaCl_2_-MgCl_2_ border, with the lowest values at the highest KCl and intermediate CaCl_2_ and MgCl_2_. These patterns suggest a synergistic effect of divalent cations, although no comparable data are available. These effects were more pronounced than in plain green Spanish-style olives [25] and natural olives [26], likely due to differences in olive firmness and diffusion properties. Moisture control is important for producers because excessive weight losses increase costs.

Lactic acid in olive moisture showed limited variations because it was adjusted to a target equilibrium level in the packaged products. Despite this, its changes were still related to salt composition. The highest values occurred at high levels of CaCl_2_, with KCl and MgCl_2_ also contributing. This effect may be linked to Ca-phenol complex formation and proton release [41].

Among colour parameters, only the colour index (CI) was affected by salt composition. CI closely followed lactic acid trends, indicating a strong relationship between the two. Lower pH likely reduced phenol oxidation [42]. This minimal impact aligns with the changes observed in whole green Spanish-style Manzanilla olives, where colour shifts could not be attributed to added salt levels [25].

From the sensory perspective, the most affected attributes were *bitter*, *hardness*, *fibrousness*, and *crunchiness*. *Bitter* scores increased with higher CaCl_2_ proportions. The lowest value occurred in the absence of CaCl_2,_ even at high levels of KCl and MgCl_2_ (CaCl_2_ vertex). This suggests that CaCl_2_ was the main driver of bitterness. This result contrasts with findings in ripe olives, where calcium showed no effect [39], possibly due to the lower phenolic content.

*Hardness*, *fibrousness*, and *crunchiness* followed similar trends. Scores increased with CaCl_2_ proportion, while KCl and MgCl_2_ had minor effects. This indicates that CaCl_2_ was the dominant factor. Its effect is likely related to interactions with organic olive components and the pimento-paste gel. Calcium forms stable complexes with pectic polysaccharides, restoring firmness lost during alkaline processing [43]. Previous studies on calcium-mediated gelation of olive pectic extracts support this mechanism. Studies on the rheological properties of pectic exudates (OPE)/calcium systems as a function of galacturonic acid (Ga1A) and calcium concentrations showed that Ga1A and calcium concentrations were critical for gelation [44]. The OPE/calcium gels exhibit a power-law dependence on calcium concentration, with the polymer concentration dependence higher than that observed for other pectic origins. The added calcium can be incorporated into such arabinan-rich pectic polysaccharide structures, forming dimeric calcium complexes that enhance their cohesiveness [44]. The addition of calcium chloride, acetate, and lactate also increased the firmness of Iranian black olive cultivars and the kinaesthetic sensations [45].

Magnesium, although structurally similar to calcium, showed little effect on texture. Magnesium is part of the green olive chlorophyll and, during processing, undergoes a pheophytinisation reaction, with progressive substitution of the Mg^2+^ by 2H^+^. This transformation may explain its losses along processing [46] and its lack of involvement in texture-related components.

The strong correlation among *hardness*, *fibrousness*, and *crunchiness* makes it challenging to distinguish among these attributes. Similar findings have been reported in other salt-replacement studies during fermentation [19,25]. Future studies should consider prioritizing these relationships, especially in consumer-based tests.

The similarity between the *overall score* and *purchasing predisposition* was expected. *Purchasing predisposition* was slightly lower due to greater caution when making purchase decisions. Products with high KCl and balanced CaCl_2_ and MgCl_2_ levels were preferred. PLS-R analysis showed that *appearance* and *odour*/*smell* positively influenced acceptance. In contrast, bitter, kinaesthetic characteristics, and *salty* and *acid* negatively impacted responses. *Bitter* was the only attribute with a negative association with both the *overall score* and *purchasing predisposition*. This adverse effect of bitterness in product acceptance has been consistently reported across olive table olive cultivars and processing methods [25,27].

However, the relationship between calcium and bitterness in table olives has not yet been studied in depth. Several bitter compounds have been identified in olives through correlation studies. These include oleuropein, oleuropein aglycone, ligstroside aglycone, oleacin, oleocanthal, elenolic acid, and elenolic acid methyl ester, all of which are strongly associated with bitterness [47]. A direct reaction between calcium and phenolic compounds is unlikely. Instead, calcium is more likely to interact with carbohydrate components of the olive tissue, thereby increasing tissue rigidity and texture [39]. This structural straightening may hinder the diffusion of bitter phenolics from the pulp, leading to higher perceived bitterness in stuffed olives. In addition, recent studies on the activation of specific bitter taste receptors by olive oil phenolics [48] suggest that bitterness could eventually be predicted and controlled by monitoring key compounds, such as oleuropein aglycone and ligstroside aglycone.

An increase in bitterness associated with calcium incorporation has also been reported in other fermentation studies. For example, cracked olives of the *Manzanilha Algarvia* cultivar fermented in 8% NaCl brine or in mixtures of potassium and calcium chloride showed acceptable sensory quality only when NaCl or a 50% NaCl–KCl mixture was used. Treatments containing 2.7–4.0% CaCl_2_ were considered unacceptable [49]. In contrast, in Spanish-style and Castelvetrano olives of *the Nocellara del Belice* cultivar, bitterness was linked to the presence of KCl rather than calcium [50]. Moreover, Mastralexi et al. [51] reported good consumer acceptance when substituting 50% of NaCl with other chloride salts in Spanish-style PDO *Elies Halkidikis* olives. Conversely, other authors have observed that calcium did not increase bitterness or cause discoloration [44]. Taken together, these contrasting findings indicate that bitter taste in fortified olives remains a complex and unresolved issue.

Cluster analysis further indicated that both physicochemical and sensory characteristics of the new products were entirely dissimilar to those in the Control. Then, fortification produced a presentation that was completely different from those actually in the market (Figure 6).

Cluster analysis showed that fortified products clearly differed from the commercial Control, forming a distinct group with characteristic physicochemical and sensory profiles. However, a broader interpretation of the relationships between physicochemical properties and sensory attributes was obtained through PLS-R. In this case, the *overall score* and *purchasing predisposition* aligned more closely with KCl, combined acidity, and lactic acid. *Appearance* was strongly related to colour parameters, CaCl_2_, and kinaesthetic attributes. The analysis also emphasizes the nearly neutral role of MgCl_2_ on sensory attributes, which may be advantageous for producers considering magnesium fortification. Among the treatments, R6, R8, and R10 showed the most favourable sensory positioning. Nevertheless, these models were developed under specific conditions, and their extrapolations to other products would require appropriate adjustments.

One key advantage of using RSM is its ability to identify optimal salt combinations under predefined constraints. The solution obtained in this study represents a compromise between favourable and unfavourable characteristics and is only one example of several possible outcomes within the experimental design. The model suggested a high KCl concentration (13.16 g/L), which positively affected its acceptance. Increasing CaCl_2_ improved firmness-related attributes but also enhanced *bitter* taste. While RSM allows direct prediction of formulation effects, it is inherently limited to the consumers’ preferences represented in this study. Even so, the study provides the flexibility to adapt formulations to different production goals or market requirements.

Overall, the results indicate that pimento-paste stuffed green Spanish-style olives and similar products can be successfully developed. KCl appears to be a favourable salt for sensory acceptance, while MgCl_2_ shows a broadly neutral effect. CaCl_2_ improves *firmness*, *hardness*, *fibrousness*, and *crunchiness* but must be used cautiously due to its impact on bitterness. Achieving an appropriate balance among these salts is therefore essential. These findings support the development of reduced-sodium table olives and align with growing interest in products that combine improved nutritional profiles with acceptable sensory quality [52].

## 5. Conclusions

The present study demonstrated, using RSM, that the production of mineral nutrient-fortified pimento-paste stuffed olives with similar or even improved quality characteristics is feasible. From a technological and sensory perspective, KCl exerted predominantly beneficial effects, whereas MgCl_2_ showed a limited influence. Although CaCl_2_ enhanced texture-related attributes such as *hardness*, *crunchiness*, and *fibrousness*, its technological benefits were partially offset by an increase in *bitter* taste, suggesting that its use must be carefully controlled.

From a nutritional standpoint, the partial replacement of NaCl with KCl and MgCl_2_ effectively reduced sodium while enhancing the product’s mineral profile, thereby supporting the use of these formulations as a strategy for nutritional fortification. Based on the RSM optimization, a formulation containing 13.16 g/L of KCl and 4.41 g/L of MgCl_2_ is recommended for health-oriented products, as it balances improved mineral intake with acceptable quality and sensory attributes. For market segments prioritizing texture, moderate CaCl_2_ supplementation may be considered, provided that its potential sensory drawbacks are considered.

It must be emphasized that these conclusions are derived from empirical statistical models and describe predictive relationships rather than mechanistic phenomena. Furthermore, the models’ validity is restricted to the specific experimental conditions investigated in this work, and extrapolation beyond these conditions should be performed with caution.

## Figures and Tables

**Table 1:** Packaging of pimento-paste stuffed green Spanish-style olives in KCl, CaCl_2_, and MgCl_2_ fortified brines—D-optimum mixture cubic model used as experimental design.

Design Points	Expected Chloride Salt Contents in the Packaged Product (g/L)			Expected Chloride Salt Contents in the Packaged Product Expressed in Actual Units (g/L)		
	KCl	CaCl_2_	MgCl_2_	KCl	CaCl_2_·2H_2_O	MgCl_2_·6H_2_O
Run 1	5	10	10	10	25	31
Run 2	15	10	0	29	25	31
Run 3	15	0	10	29	0	0
Run 4	5	10	10	10	25	31
Run 5	10	5	10	20	13	16
Run 6	15	5	5	29	13	16
Run 7	8.33	8.33	8.33	16	21	26
Run 8	15	0	10	29	0	0
Run 9	15	10	0	29	25	31
Run 10	13.33	3.33	8.33	26	8	10
Run 11	13.33	8.33	3.33	26	21	26
Run 12	11.67	6.67	6.67	23	17	21
Run 13	10	5	10	20	13	16
Run 14	10	10	5	20	25	31

Note: These values correspond to the targeted equilibrium concentrations in the packaged product. The actual levels in packaging brines accounted for the salt’s hydration state and the ratio of stuffed olives to brine in the containers. At the right are the expected values, expressed in the actual levels of the real hydration degree of the salts and the packaging characteristics (brine: 130 mL; olives: 170 g; stuffed olive moisture: 72.96%). Notice that actual concentrations are valid only for the conditions of this experiment.

**Table 2:** Packaging of pimento-paste stuffed green Spanish-style olives in KCl, CaCl_2_, and MgCl_2_ fortified brines—characteristics of the stuffing material and the stuffed olives used as raw materials in the study.

Parameter	Average (Standard Error)	LCI (95%)	HCI (95%)
Stuffed olives *			
Weight (g)	3.15 (0.08)	3.01	3.30
Diameter (cm)	1.56 (0.02)	1.54	1.60
High (cm)	1.77 (0.02)	1.73	1.80
Volume (mL)	3.15 (0.05)	3.06	3.24
Stuffing material			
Weight (g)	0.49 (0.01)	0.47	1.57
Volume (mL)	0.34 (0.02)	0.31	0.34
Stuffed olive-derived features			
Entire stuffed olives density (g/mL)	0.999 (0.010)	0.975	1.02
Stuffing material density (g/mL)	1.49 (0.04)	1.42	1.57
Stuffing material proportion (%)	15.77 (0.46)	15	16.59
Olive pulp proportion (%)	84.20 (0.46)	83.41	85.00
Stuffed olives moisture (%)	72.96 (0.50)	71.97	73.95
Stuffing material moisture (%)	86.90 (0.02)	86.86	89.94
Olive pulp moisture (%)	68.49 (0.14)	68.21	68.67

Note: * olive plus stuffing material. LCI, lower confidence interval. HCI, higher confidence interval.

**Table 3:** Packaging of pimento-paste stuffed green Spanish-style olives in brines fortified with KCl, CaCl_2_, and MgCl_2_. Physicochemical properties of the storage brine, desalting solutions (aiming for 2.5% NaCl in the stuffed olive moisture), and brines after packaging.

Treatments	pH	Titratable Acidity (g/L)	Combined Acidity (mEq/L)	Estimated Lactic Acid (g/L) ^a^	HPLC Lactic Acid (g/L)
Storage brine	3.90 (0.03)	9.20 (0.40)	101.6 (3.4)	18.34 (0.09)	17.45 (0.29)
Desalting solution	3.97 (0.06)	2.95 (0.25)	37.3 (1.4)	6.30 (0.13)	5.97 (0.05)
Treatment brines					
Run 1	3.58 (0.04)	2.45 (0.05)	20.9 (0.2)	4.33 (0.03)	4.37 (0.08)
Run 2	3.63 (0.08)	2.40 (0.10)	24.0 (1.0)	4.56 (0.01)	4.46 (0.06)
Run 3	3.94 (0.06)	2.05 (0.15)	24.8 (1.1)	4.28 (0.05)	4.40 (0.06)
Run 4	3.54 (0.03)	2.45 (0.05)	22.5 (0.3)	4.47 (0.07)	4.35 (<0.01)
Run 5	3.67 (0.03)	2.35 (0.05)	23.1 (0.5)	4.42 (<0.01)	4.33 (<0.01)
Run 6	3.63 (0.02)	2.30 (<0.01)	24.2 (0.3)	4.48 (0.03)	4.34 (0.05)
Run 7	3.66 (0.06)	2.30 (<0.01)	23.6 (0.5)	4.42 (0.04)	4.36 (0.04)
Run 8	3.59 (0.11)	2.40 (0.10)	23.5 (0.6)	4.52 (0.04)	4.36 (0.06)
Run 9	3.62 (0.03)	2.35 (0.05)	23.3 (0.3)	4.45 (0.07)	4.34 (0.04)
Run 10	3.62 (0.07)	2.40 (0.10)	23.5 (0.1)	4.51 (0.10)	4.37 (0.02)
Run 11	3.67 (0.03)	2.40 (<0.01)	23.4 (0.3)	4.51 (0.02)	4.39 (0.01)
Run 12	3.69 (0.03)	2.30 (<0.01)	23.8 (0.3)	4.44 (0.03)	4.42 (0.01)
Run 13	3.30 (0.04)	2.50 (<0.01)	22.1 (0.6)	4.49 (0.06)	4.36 (0.02)
Run 14	3.56 (0.02)	2.40 (<0.01)	22.8 (0.2)	4.46 (0.01)	4.46 (0.06)
Control ^b^	3.74 (0.01)	2.30 (<0.01)	24.7 (0.3)	4.52 (0.03)	4.43 (0.09)

Note: ^a^ Lactic acid estimated considering combined acidity plus titratable acidity. ^b^ Control, packaged under standard industrial conditions. HPLC, lactic acid determined by high-performance liquid chromatography (HPLC). NaCl in the pimento-paste stuffed olive storage solution, 73.2 (0.3) g/L; NaCl in the desalting brine, 25 (0.02) g/L. Values are average with standard error in parentheses.

**Table 4:** Packaging of pimento-paste stuffed green Spanish-style olives in KCl, CaCl_2_, and MgCl_2_ fortified brines—physicochemical parameters of stored stuffed olives, desalted product, and packaged stuffed olives.

Treatments	Firmness ^b^ (N/g)	Moisture ^b^ (g/100 g Olives)	Lactic Acid in Moisture ^b^ (g/L)
Stored stuffed olives	16.12 (5.10)	72.96 (0.50)	17.95 (0.34)
Desalted stuffed olives	14.22 (4.50)	75.72 (0.07)	6.89 (0.10)
Packaged olives			
Run 1	14.32 (4.53)	73.69 (0.05)	4.42 (0.01)
Run 2	15.44 (4.88)	75.63 (0.05)	4.44 (0.04)
Run 3	11.63 (3.68)	75.43 (0.03)	4.31 (0.03)
Run 4	15.18 (4.80)	74.16 (0.11)	4.34 (0.02)
Run 5	16.02 (5.07)	75.23 (0.10)	4.34 (0.05)
Run 6	16.65 (5.27)	73.82 (0.20)	4.31 (0.01)
Run 7	15.38 (4.86)	74.69 (0.13)	4.41 (0.05)
Run 8	12.16 (3.85)	74.86 (0.09)	4.34 (<0.01)
Run 9	14.49 (4.58)	75.04 (0.03)	4.43 (0.02)
Run 10	13.15 (4.16)	74.22 (0.39)	4.36 (0.06)
Run 11	17.18 (5.43)	74.23 (0.45)	4.41 (0.08)
Run 12	14.40 (4.55)	74.76 (0.05)	4.42 (0.10)
Run 13	14.51 (4.59)	74.03 (0.11)	4.29 (0.01)
Run 14	16.04 (5.07)	74.13 (0.26)	4.43 (0.02)
Control ^a^	12.43 (3.93)	75.06 (0.29)	4.39 (0.04)

Notes: ^a^ Control, packaged under standard industrial conditions. ^b^ Data refers to the entire stuffed olive (olive pulp plus stuffing material). NaCl in the storage solution, 73.2 (0.3) g/L; NaCl in the desalting brine, 25 (0.02) g/L. Data refer to average values and standard errors in parentheses.

**Table 5:** Packaging of pimento-paste stuffed green Spanish-style olives in brines fortified with KCl, CaCl_2_, and MgCl_2_—color parameters of stored, desalted, and packaged stuffed olives.

Treatment	CI	L*	a*	b*	*Ch*	h*	(−a*/b*)
St. stuffed olives	25.11 (0.35)	49.62 (0.06)	4.92 (0.07)	32.97 (0.01)	33.34 (<0.01)	81.51 (0.12)	−0.149 (0.002)
Des. stuffed olives	25.41 (0.38)	50.23 (0.11)	4.56 (0.17)	33.09 (0.30)	33.40 (0.31)	82.16 (0.21)	−0.138 (0.004)
Pac. stuffed olives							
Run 1	25.27 (0.16)	49.95 (0.11)	4.48 (0.01)	32.38 (0.42)	32.69 (0.42)	82.12 (0.08)	−0.138 (0.001)
Run 2	23.78 (0.23)	48.81 (0.36)	4.38 (0.04)	31.02 (0.40)	31.33 (0.40)	81.97 (0.04)	−0.141 (0.001)
Run 3	23.72 (0.05)	48.45 (0.18)	4.32 (0.02)	31.24 (0.08)	31.54 (0.08)	82.13 (0.06)	−0.138 (0.001)
Run 4	25.88 (0.44)	50.35 (0.11)	4.58 (0.07)	31.77 (0.05)	32.10 (0.06)	81.80 (0.11)	−0.144 (0.002)
Run 5	25.03 (0.14)	49.19 (0.04)	4.86 (0.07)	32.28 (0.38)	32.64 (0.36)	81.44 (0.21)	−0.150 (0.004)
Run 6	26.05 (0.25)	50.67 (0.23)	4.47 (0.05)	32.28 (0.15)	32.59 (0.15)	82.12 (0.04)	−0.138 (0.001)
Run 7	24.41 (0.31)	50.45 (0.15)	4.09 (0.08)	32.86 (0.56)	33.11 (0.56)	82.91 (0.01)	−0.124 (<0.001)
Run 8	24.77 (0.15)	49.89 (0.23)	4.46 (0.06)	31.95 (0.16)	32.25 (0.16)	82.05 (0.07)	−0.140 (0.001)
Run 9	23.84 (0.12)	50.29 (0.04)	4.43 (0.01)	31.43 (0.06)	31.74 (0.06)	81.98 (<0.01)	−0.141 (<0.001)
Run 10	24.36 (0.56)	49.82 (0.50)	4.22 (0.12)	32.07 (0.43)	32.35 (0.44)	82.51 (0.11)	−0.132 (0.002)
Run 11	24.44 (0.22)	50.09 (0.06)	4.39 (0.02)	31.48 (0.35)	31.78 (0.34)	82.06 (0.05)	−0.139 (0.001)
Run 12	23.94 (0.35)	49.50 (0.32)	4.18 (<0.01)	30.95 (0.64)	31.23 (0.63)	82.31 (0.16)	−0.135 (0.003)
Run 13	25.23 (0.01)	49.96 (0.17)	4.53 (0.04)	31.99 (0.29)	32.30 (0.29)	81.95 (0.01)	−0.141 (<0.001)
Run 14	24.00 (0.46)	49.57 (0.36)	4.29 (0.01)	31.21 (0.33)	31.50 (0.33)	82.18 (0.09)	−0.137 (0.002)
Control ^a^	23.75 (0.07)	49.88 (0.28)	3.86 (0.10)	32.06 (0.14)	32.29 (0.13)	83.14 (0.20)	−0.120 (0.003)

Notes: ^a^ Control, packaged under standard industrial conditions. Data are average values with standard error in parentheses.

**Table 6:** Packaging of pimento-paste stuffed green Spanish-style olives in brines fortified with KCl, CaCl_2_, and MgCl_2_. Table includes average values of sensory attributes, *overall score*, and *purchasing predisposition*, across treatments.

Treatments	*Appearance*	*Odour*/ *Smell*	*Acid*	*Bitter*	*Salty*	*Hardness*	*Fibrousness*	*Crunchiness*	*Overall* *Score*	*Purchasing* *Predisposition*
Run 1	6.70 (0.18)	6.06 (0.18)	5.30 (0.20)	6.98 (0.19)	6.74 (0.20)	6.05 (0.16)	6.06 (0.15)	5.97 (0.16)	5.10 (0.18)	4.47 (0.19)
Run 2	6.32 (0.18)	5.64 (0.20)	5.24 (0.19)	5.94 (0.19)	6.53 (0.17)	6.32 (0.15)	6.31 (0.16)	6.00 (0.16)	5.56 (0.18)	5.16 (0.20)
Run 3	6.09 (0.19)	5.85 (0.17)	5.00 (0.19)	5.56 (0.19)	6.27 (0.20)	5.15 (0.17)	5.30 (0.17)	5.03 (0.18)	5.09 (0.19)	4.58 (0.20)
Run 4	6.42 (0.16)	5.83 (0.16)	5.34 (0.20)	6.99 (0.20)	6.55 (0.20)	6.52 (0.15)	6.42 (0.16)	6.28 (0.16)	4.64 (0.21)	4.18 (0.21)
Run 5	6.48 (0.16)	5.87 (0.16)	5.34 (0.19)	6.20 (0.20)	6.40 (0.19)	6.04 (0.16)	5.99 (0.16)	6.07 (0.17)	5.34 (0.18)	4.74 (0.21)
Run 6	6.72 (0.17)	6.33 (0.19)	5.16 (0.20)	5.69 (0.20)	6.27 (0.17)	5.78 (0.16)	5.98 (0.18)	5.61 (0.17)	6.07 (0.18)	5.63 (0.20)
Run 7	6.65 (0.18)	5.68 (0.17)	5.13 (0.19)	6.66 (0.20)	6.08 (0.20)	6.08 (0.15)	6.07 (0.16)	5.80 (0.16)	5.17 (0.19)	4.62 (0.20)
Run 8	6.52 (0.18)	6.09 (0.18)	5.14 (0.19)	5.66 (0.20)	6.18 (0.17)	5.15 (0.16)	5.11 (0.16)	4.83 (0.17)	5.62 (0.21)	5.23 (0.23)
Run 9	6.14 (0.17)	5.82 (0.18)	5.34 (0.20)	6.19 (0.18)	6.49 (0.19)	6.19 (0.16)	6.17 (0.16)	5.88 (0.18)	5.31 (0.16)	4.74 (0.18)
Run 10	6.59 (0.19)	6.01 (0.16)	5.35 (0.17)	5.91 (0.20)	6.47 (0.17)	5.86 (0.16)	5.90 (0.17)	5.81 (0.18)	5.47 (0.18)	5.05 (0.20)
Run 11	6.97 (0.18)	6.62 (0.18)	5.54 (0.21)	6.26 (0.20)	6.44 (0.19)	6.23 (0.16)	6.14 (0.16)	5.94 (0.17)	6.04 (0.16)	5.62 (0.18)
Run 12	6.67 (0.17)	6.22 (0.17)	5.31 (0.20)	6.09 (0.21)	6.25 (0.19)	5.96 (0.18)	5.80 (0.19)	5.61 (0.18)	5.78 (0.18)	5.26 (0.21)
Run 13	6.64 (0.17)	5.98 (0.19)	5.42 (0.21)	6.63 (0.21)	6.22 (0.20)	6.10 (0.17)	5.97 (0.16)	5.87 (0.17)	5.51 (0.20)	5.13 (0.21)
Run 14	6.57 (0.16)	5.75 (0.16)	5.22 (0.22)	6.77 (0.22)	6.35 (0.21)	6.21 (0.17)	6.26 0.17)	6.02 (0.17)	5.11 (0.19)	4.62 (0.21)
Control ^a^	6.74 (0.17)	6.15 (0.20)	5.04 (0.21)	4.40 (0.20)	6.25 (0.20)	5.10 (0.17)	5.03 (0.17)	5.01 (0.18)	6.12 (0.19)	5.73 (0.21)

Notes: ^a^ Control packaged under current industrial conditions. Data are average values with standard error in parentheses.

**Table 7:** Packaging of pimento-paste stuffed green Spanish-style olives in brines fortified with KCl, CaCl_2_, and MgCl_2_—correlation between sensory attribute scores (including *overall score* and *purchase predisposition*) from consumer panel.

	*Appearance*	*Odour*/ *Smell*	*Acid*	*Bitter*	*Salty*	*Hardness*	*Fibrousness*	*Crunchiness*	*Overall Score*	*Purch. Predis.*	Average	SD
*Appearance*	1.000	0.693 *	0.32	0.027	−0.125	0.071	0.007	0.08	0.543 *	0.532 *	6.55	0.23
*Odour*/*Smell*	0.693 *	1.000	0.313	−0.274	−0.039	−0.215	−0.247	−0.228	0.714 *	0.693 *	5.99	0.26
*Acid*	0.32	0.313	1.000	0.548 *	0.459	0.712 *	0.624 *	0.698 *	0.006	<0.0001	5.26	0.15
*Bitter*	0.027	−0.274	0.548 *	1.000	0.372	0.790 *	0.780 *	0.770 *	−0.666 *	−0.678 *	6.13	0.67
*Salty*	−0.125	−0.039	0.459	0.372	1.000	0.487	0.513 *	0.564 *	−0.324	−0.35	6.37	0.17
*Hardness*	0.071	−0.215	0.712 *	0.790 *	0.487	1.000	0.975 *	0.967 *	−0.371	−0.378	5.92	0.44
*Fibrousness*	0.007	−0.247	0.624 *	0.780 *	0.513 *	0.975 *	1.000	0.959 *	−0.395	−0.406	5.9	0.42
*Crunchiness*	0.08	−0.228	0.698 *	0.770 *	0.564 *	0.967 *	0.959 *	1.000	−0.408	−0.426	5.72	0.43
*Overall score*	0.543 *	0.714 *	0.006	−0.666 *	−0.324	−0.371	−0.395	−0.408	1.000	0.989 *	5.46	0.42
*Purch. Predis.*	0.532 *	0.693 *	<0.001	−0.678 *	−0.35	−0.378	−0.406	−0.426	0.989 *	1.000	4.98	0.47

Notes: * significant at *p*-value ≤ 0.05 n =15. *Purch. Predis.*, *purchase predisposition*.

## Data Availability

The original contributions presented in the study are included in the article, further inquiries can be directed to the corresponding author.
